# Editorial: Cognitive Mechanisms for Safe Road Traffic Systems

**DOI:** 10.3389/fnrgo.2022.897659

**Published:** 2022-04-25

**Authors:** Giovanni Vecchiato, Christer Ahlström, Lewis L. Chuang

**Affiliations:** ^1^Institute of Neuroscience, National Research Council of Italy, Parma, Italy; ^2^Swedish National Road and Transport Research Institute (VTI), Linköping, Sweden; ^3^Department of Biomedical Engineering, Linköping University, Linköping, Sweden; ^4^Department Humans and Technology, Institute for Media Research, Faculty of Humanities, Chemnitz University of Technology, Chemnitz, Germany

**Keywords:** automated driving, driving behavior, road safety, brain signals, eye tracking, EMG, autonomic signals

Human behavior is often cited as the primary contributing factor to road accidents—over 90% of all crashes are attributed to “human error” (Singh, 2015). This implicitly suggests that accidents could be avoided if only drivers behaved better and has, thus, fuelled enthusiasm for (semi-)automated vehicles, which do not suffer from human frailty and are more likely to follow the rules. Nonetheless, this perspective is flawed. First, most road users strive to avoid road accidents. Second, fatalities persist even with (semi-)automated vehicles and it remains unclear if increased adoption of more automation will change the situation at all (Mueller et al., 2021). Modern perspectives suggest that “human error” is a product of not only individual behavior but the system that we operate within (Read et al., 2021). An individual cannot be understood without taking into account their relationship with the working environment. Safe vehicles are those that enable drivers to act with a minimal margin for unintended error while ensuring that road traffic systems cater to user autonomy. Even if automation can mitigate driving-related risks, it will simply present new challenges that can only be anticipated by first understanding the cognitive mechanisms associated with operating in road traffic systems. To this aim, it is vital that we possess better tools to understand, measure and monitor human behavior and the corresponding cerebral activity across diverse road scenarios, including those that do not generate overt behavior.

This Research Topic invited manuscripts that covered modeling, behavioral and neurophysiological measures investigating conventional and (semi-)automated driving with the goal to develop a safe human-centric road traffic system. The submitted contributions responded to this challenge in various ways, including state-of-the-art physiological measures to assess the driver's mental state, theoretical and empirical methodological approaches to advance the present knowledge, and challenges in vehicle automation.

Several papers in this article Research Topic emphasize that fatigue, cognitive load, inattention, and stress are multidimensional constructs that must be interpreted within a context, taking both endogenous and exogenous factors into account. This is important not only for accurate estimation of the driver's state, but also when selecting appropriate countermeasures.

Here, the review by Chong and Baldwin addresses the underlying mechanisms of fatigue and how different types of fatigue arise. For example, active fatigue is related to long-term neuronal potentiation and local sleep, whereas passive fatigue disturbs the interplay between the dorsal attention network and the default mode network. Circadian effects can further moderate both passive and active fatigue, linking the two to a third category of fatigue, namely sleep-related fatigue. Discriminating for different types of fatigue is a crucial first step for adopting appropriate countermeasures: active fatigue should be countered by reduced task demand, passive fatigue by increased task demand, and sleep-related fatigue by sleep and recuperation.

The effects of task demand on cognitive load is addressed by Nilsson et al. Their approach accounts for the many complexities that arise when estimating cognitive load in a realistic situation, such as traffic. First, they characterize cognitive load as a multidimensional construct that consists of many mental responses to added task demand. Second, they highlight that cognitive load does not occur in isolation but is part of a complex response to task demands in a specific context. Finally, physiological measures typically correlate with more than one mental state, thus limiting the inferences that can be made from any individual state.

Kerautret et al. provide a systematic review that guides the selection of appropriate physiological measures for quantifying acute stress. Once again, physiological response to a driver state change is characterized as a multidimensional construct, where several measures, including heart rate, R-R intervals and pupil diameter, respond to driver stress levels. When aiming to improve the practical usefulness of stress detection devices, it is important to start considering the context where the stress level is measured. Increased sympathetic activity is a reasonable response to a critical event or complex unfamiliar environments, and it is important to realize that a temporarily elevated stress level is not necessarily a bad thing.

What is appropriate and inappropriate behavior? More importantly, Ahlström et al. demonstrate that this deceptively simple classification depends on context in this article that describes eye movements and how they relate to attentive driving. Essentially, it is not just where drivers look, but also why and what else they can see and where they do not look. The authors suggest a glance analysis approach that classifies glances based on their purpose, thus accounting for context or motivation behind eye movements.

The present article Research Topic also comprises a series of theoretical and empirical works that leverage methodological aspects to foster the knowledge on fundamental brain processes for driving research.

Kujala and Lappi outline a predictive processing approach for studying attentional demand and inattention in driving, based on neuro-inspired theories of uncertainty processing and experimental research that combine brain imaging, visual occlusion and computational modeling. This approach improves the definition and detection of inattentive driving as a step toward designing attention monitoring systems for conventional and semi-automated driving.

Vecchiato provides a perspective on the technological maturity of hybrid systems, which could improve the predictive power of a single neurophysiological measurement. Electroencephalography (EEG) is often constrained by robustness, comfortability, and high data variability affecting the decoding performance in driving scenarios. Hence, additional peripheral signals can be combined with EEG for increasing replicability and the overall performance of the brain-based action decoder.

Getzmann et al. explored the ecological and internal validity of round-the-ear electrodes (cEEGrids) measurements. Their longitudinal study returns consistent modulations in the alpha and theta bands, along with driving speed and steering wheel angular velocity reflecting the complexity of the driving task between the two measurements. Overall, the reliability and ecological validity of cEEGrid electrodes were satisfactory in the context of driving-related parameters.

Among the many issues related to the safe use of driving automation, attention drift due to the modulation of internal sources could play an important role in the emergence of out-of-the-loop (OOTL) situations and associated performance problems (Merat et al., 2019).

Gouraud et al. address the possibility of the gradual emergence of attentional decoupling and the differences created by the sensory modality used to convey targets using EEG measurements. Their results underline the complex influence of perceptual decoupling on operators' behavior and EEG measures.

Sensory skills can be augmented through training and technological support. Exploiting this, Sakai et al. used fMRI to compare brain responses to auditory cues for self-localization, modulated by a sensory augmentation training in a simulated driving environment. Their results suggest that the use of auditory cues for self-localization during locomotion relies on multimodality in higher-order somatosensory, rather than visual, areas.

The complexity of autonomous navigation increases due to the lack of road signs and pedestrians' presence. The work by Petit et al. deals with the perception of collision risk from the viewpoint of a passenger sitting in the driver's seat. Such users delegate total control of their vehicle to an autonomous system and this article investigated the subjective risk assessment with a system based on the measurement of the electrodermal activity. The results demonstrate that reducing safety margins increases risk perception.

Considerable evidence suggest that humans may interact differently with autonomous vehicles (AVs) as compared to human-driven vehicles (HVs). Unni et al. investigated whether participants would value interactions with AVs differently compared to HVs, and if these differences can be characterized in terms of behavior and brain responses. Using hemodynamic response features from whole-head fNIRS, they could predict whether participants decided to turn in front of HVs or AVs in the decision-making phase. The insights provided here may be useful for developing driver assistance systems to assess interactions in future mixed traffic environments involving AVs and HVs.

Finally, Fredriksson et al. provide a roadmap for the development of future Occupant Status Monitoring (OSM) in the EuroNCAP protocol. This considers a range of known and emerging safety risks, including driving while intoxicated by alcohol or drugs, cognitive distraction, and the driver engagement requirements for supervision and take-over performance with assisted and automated driving features.

The works collected by this Research Topic describe a wide range of challenges that have to be addressed as we improve on our knowledge of cognitive mechanisms, relevant for the design of safe road traffic systems. In view of the strong interest in the academic and industrial fields, we believe that the present Research Topic will increase rigor and reproducibility in driving research.

## Author Contributions

All authors listed have made a substantial, direct, and intellectual contribution to the work and approved it for publication.

## Conflict of Interest

The authors declare that the research was conducted in the absence of any commercial or financial relationships that could be construed as a potential conflict of interest.

## Publisher's Note

All claims expressed in this article are solely those of the authors and do not necessarily represent those of their affiliated organizations, or those of the publisher, the editors and the reviewers. Any product that may be evaluated in this article, or claim that may be made by its manufacturer, is not guaranteed or endorsed by the publisher.
